# Increased Intake and Use of Euthanasia in Dog Shelters in the UK and Republic of Ireland 2021–2023

**DOI:** 10.3390/ani16050791

**Published:** 2026-03-03

**Authors:** Helena Hale, Paige McCormack, Siobhan Mullan

**Affiliations:** 1 Bristol Veterinary School, University of Bristol, Langford, Bristol BS40 5DU, UK; 2 University Centre Reaseheath, Reaseheath College, Nantwich CW5 6DF, UK; paige.mccormack@reaseheath.ac.uk; 3 School of Veterinary Medicine, University College Dublin, D04 V1W8 Dublin, Ireland; siobhan.mullan@ucd.ie

**Keywords:** dog shelter, freedom of information request, stray dog population, dog euthanasia, Local Authority

## Abstract

In the United Kingdom (UK) and Republic of Ireland (ROI), Local Authorities (LAs) are responsible for stray dogs, with close cultural links and trade in dogs between these jurisdictions. However, there is a lack of coordinated data on the annual numbers of dogs entering LA shelters, reasons for their intake and outcomes across these countries. By making a Freedom of Information (FOI) request to all LAs in the UK and ROI, this study aimed to elucidate and combine this information for 2021–2023, including euthanasia figures and policies relating to animal welfare. In total, 319 of the 403 LAs responded and 254 (63%) provided usable data for the three years. In all countries combined, the numbers of dogs entering LA shelters increased from 16,310 in 2021 to 21,408 in 2022 and to 23,287 in 2023. The proportion of dogs taken into shelter by LAs that were then rehomed directly or via a rehoming organisation increased from 41% in 2021 to 53% 2023, but so did the proportion of dogs euthanased, except in Scotland. Overall, 6.3% of LA kennelled stray dogs were euthanased in 2023, compared with 3.0% in 2022 and 1.9% in 2021. The most commonly euthanased breed type in 2021 was the Staffordshire Bull Terrier and by 2023, other Bull breeds, XL Bullies (from 1 case in 2021 to 150 cases in 2023 in responding LAs), and Collies were being euthanased more frequently. Written welfare and euthanasia policies were reportedly present for 27% and 23% of Las, respectively. A harmonised approach to LA dog data is recommended to enable monitoring over time and between LAs, regions, and countries.

## Simple Summary

In the United Kingdom (UK) and Republic of Ireland (ROI), Local Authorities (LAs) are responsible for stray dogs, with close cultural links and trade in dogs between these jurisdictions. However, there is a lack of coordinated data on the annual numbers of dogs entering LA shelters, reasons for their intake and outcomes across these countries. By making a Freedom of Information (FOI) request to all LAs in the UK and ROI, this study aimed to elucidate and combine this information for 2021–2023. A total of 254 LAs (63%) provided usable data for the three years. The number of dogs entering LA shelters across all countries increased from 16,310 in 2021 to 21,408 in 2022 and to 23,287 in 2023. The proportion of dogs taken into shelter by LAs that were rehomed increased from 41% in 2021 to 53% in 2023. Notably, 6.3% of LA kennelled stray dogs were euthanased in 2023, much increased from the 3.0% in 2022 and 1.9% in 2021. The most commonly euthanased breed type in 2021 was the Staffordshire Bull Terrier and by 2023, other Bull breeds, XL Bullies (from 1 case in 2021 to 150 cases in 2023 in responding LAs), and Collies were being euthanased more frequently. A harmonised approach to LA dog data is recommended to enable monitoring over time and between LAs, regions, and countries.

## 1. Introduction

Responsible dog ownership in the United Kingdom (UK) and Republic of Ireland (ROI) is framed to prevent stray dogs through encouraging long-term dog–owner relationships and only carefully considered breeding [1,2]. Stray dogs in the UK and ROI may live a precarious life and be at risk of hunger, injury, and disease [3]. Local authorities (LAs) in the UK and ROI are responsible for stray dogs through their dog warden services, with a statutory duty to kennel them for seven days (UK) or five days (ROI), during which time, owners can reclaim them. Thereafter, LAs may continue to provide shelter and adopt dogs out, transfer the dog to an alternative home, e.g., via a rehoming organisation, or opt for euthanasia. Dogs in kennels frequently have restricted space, social contact, cognitive stimulation and autonomy, and are at an increased risk of developing behavioural issues [4,5]. In the last two decades, the estimated population of dogs handled by local authorities in the UK has declined overall from >120,000 in 2000 to 35,000 in 2023 [6]. Likewise in the ROI, reported figures entering LA dog shelters has reduced from >12,000 in 2015 to around 7000 in 2023 [7]. However, within this overall downward trend, a short-term increase in admissions has been observed since 2021, although total numbers in 2023 remain markedly lower than historical levels [6,7].

While separate data exist regarding some elements of LA kennel activities for UK and ROI shelters, there are no combined reports allowing direct comparisons between nations, nor has anyone investigated supporting welfare and euthanasia policies associated with LA kennels. Given that the dog populations of the UK and ROI are closely linked through shared dog trade and the cultural similarities within these nations, a harmonised investigative approach is indicated. Using data obtained through Freedom of Information (FOI) requests, our study aimed to characterise population numbers, breed types, and outcomes of dogs in LA shelters across all four UK nations and the ROI, alongside training practices and LA welfare and euthanasia policies that might influence dog outcomes.

## 2. Materials and Methods

This study was granted low-risk ethical approval by the UCD Human Research Ethics Committee, approval number LS-C-24-171-Mullan. Under the UK Freedom of Information Act 2000 [8] and ROI Freedom of Information Act 2014 [9], citizens are able to make FOI requests to access information held by public authorities. The use of FOI requests is increasingly valued for gathering research data that would otherwise be inaccessible and which may help advance understanding of the topic of interest [10,11,12]. An FOI request was sent to the FOI email address for all 403 local authorities (LAs) in England (n = 308), Wales (n = 22), Scotland (n = 31), Northern Ireland (n = 11), and Republic of Ireland (n = 31) in April 2024. The request comprised questions about the welfare and outcomes of dogs in LA shelters, including euthanasia. LAs were also asked for information about staff numbers and level of staff training, and to provide written policies on welfare and euthanasia if used, as well as their most recent inspection report and any recommendations for improvements. Given the limited time that FOI officers are able to spend on a request, and in relation to other recent reports of rising numbers of dogs entering shelter kennels, we asked for data between 2021 to 2023 with the aim of capturing recent patterns over time whilst optimising response rate. Recipients were advised of the intent to analyse and publish the data. The request was worded as follows:

“For each of the last 3 years (2021, 2022, 2023), please provide the following information:

- The number of dog places that you have available at any one time in your shelter(s) or pound(s)
- The number of dogs that have spent any time in your dog shelter(s) or pound(s).
- The number of stray dogs that have entered a shelter/pound(s)
- The number of dogs that have entered a shelter/pound(s) that were seized for welfare reasons
- The number of dogs that have entered a shelter/pound(s) that were seized for human safety reasons
- The number of dogs that have entered a shelter/pound(s) that were relinquished
- The top 3 breeds of the dogs that entered the shelter/pound(s) plus amount of each breed
- The number of dogs that were rehomed directly from your shelter
- The number of dogs that were moved to another rehoming centre (e.g., run by a charity) in the UK/Ireland and abroad
- The average length of stay of dogs in your shelter/pound(s)
- The number of each type of breed of dog that were euthanized.

Currently:

- Do you have any written policies on
  - Welfare of the dogs in your shelter/pound(s), including requirements for exercise, enrichment, formal behavioural assessment? Yes/No (If yes, please provide)
  - Euthanasia of dogs in your shelter/pound(s)? Yes/No (If yes, please provide)
- Please provide your most recent inspection report including the recommendations for improvement
- Please provide the Full Time Equivalent staff and level of formal training of the staff that work in your shelter/pound(s)

Thank you for your co-operation.”

### Data Handling

The data provided by LAs were in a variety of formats (pdf, Microsoft Excel Microsoft Word, and email) and were entered into a Microsoft Excel (Microsoft® Excel® for Microsoft 365 MSO (Version 2512 Build 16.0.19530.20226) 64-bit) spreadsheet for analysis. Not all LAs provided a response to the FOI request, and of those who did, they did not all answer every question. To retain as much information as possible, different numbers of responses are reported for each question and given as a proportion of the total number of responding LAs. Questions 1 to 11 requested information for 2021–2023 and, therefore, only responses that contained data for all three years were considered complete and included in analyses. Where a response appeared obviously ambiguous, it was removed, with specific instances described, for example, as more staff than the number of dogs or rehoming figures that were higher than dog intake. Two questions requested information about breeds of dogs: question 7 asked about the top three breeds entering LA kennels and question 11 asked for euthanasia figures by breed type. For question 7, given the repetition of breeds or similar breeds appearing in the top three, and the small numbers (<10) reported for some breeds, these data were categorised into breed types for reporting along with the proportion of times they appeared in the top three. Categorisation of euthanased breed types for question 11 was necessarily different to that for the top three breeds entering kennels because of the greater variation in breed types euthanased and higher numbers of some breed types that were euthanased compared with being in the top three to enter shelters. A more detailed breakdown of breed categories for both questions are given in the Results.

## 3. Results

### 3.1. Local Authority Response Rates and Service Provision

From the 403 contacted Local Authorities (LAs), a fifth (20.8%) did not acknowledge or respond to the FOI request. Of the 319 responding LAs, 67 (21%) could not be included in the final data set either because they did not provide any data (stating that they outsourced the service and did not hold data or that they had no LA kennels) (n = 51), did not provide data for all three years (n = 15), or gave an identical response to another local LA with a shared service (n = 1). This left a final dataset of 254 LAs (63% of 403 originally contacted) who provided data for all three years for at least one question (England: 191, 62.0%; Scotland: 19, 61.3%; Wales: 13, 59.0%; NI: 9, 81.8%; ROI: 22, 70.9%). Of these, 17 LAs (seven NI, nine ROI, and one England) did not provide statistics, but gave a webpage link to their council summary statistics from which available relevant data were extracted. As detailed in the results that follow, not all 254 LAs provided an answer to every question from the FOI and the proportion of responses given are summarised.

### 3.2. Number of Dogs Entering Shelter Accommodation and Reasons for Entry

One hundred and thirty-seven (53.9%) LAs provided the number of dog places available for their service, which ranged from 0 to 400, with the mean number per LA varying between countries: England 22.8; NI 29.8; ROI 21.2; Scotland 9.7; and Wales 43.1. Thirty-three (12.9%) LAs stated that there was no set number of dog places in their provision, whilst those remaining either claimed that they did not hold these data (38, 14.9%) or did not provide an answer to the question at all (46, 18.1%). Whilst LAs had been asked for the number of dog places available at any one time, some provided a number of places far exceeding their annual intake of dogs and it is unknown how many LAs may have given erroneous answers to this question.

Two hundred and twenty-eight (89.8%) LAs provided information about the number of dogs entering their shelter kennels across all three years (see Table 1), increasing from 16,310 in 2021 (mean = 70.2, range = 0–608) to 21,408 in 2022 (mean = 95.6, range = 1–638) and to 23,287 in 2023 (mean = 103.9, range = 0–742). The remaining LAs either provided no answer or stated they did not hold this information for all or some of the three years. The increase in the number of dogs occurred year on year across all countries. LAs in England took in the most dogs overall (40,368), whilst those in NI had the greatest mean number of dogs entering per LA (236) over the three years.

Considering reasons for intake, all but one of the 254 responding LAs provided data about the number of strays entering their shelters for all three years. There was an overall increase in strays between 2021 to 2023, with a total of 15,907 (mean 65.3 per LA and range of 0–608) in 2021, 22,735 (mean 89.9 per LA and range of 1–535) in 2022, and 25,661 (mean 101.4 per LA and range of 0–742) in 2023. This pattern of increasing numbers occurred across all countries, with the most marked increase being between 2021 and 2022. Again, NI had the greatest mean number of stray dogs entering their kennels per LA (229.9).

A total of 186 (73.2%) LAs provided data for the number of dogs relinquished to their shelter for all three years, with the majority (112, 60.2%) reporting zero relinquishments. Of the 74 LAs reporting relinquishments, 1753 dogs were relinquished across all countries in 2021 (mean 9.2, range 0–192), 2593 in 2022 (mean 13.6, range 0–304), and 2649 in 2023 (mean 13.9, range 0–386), revealing an overall increase, especially between 2021 and 2022.

A total of 209 (82.3%) LAs provided data about the number of dogs seized for animal welfare reasons across all three years. However, the vast majority (173, 82.8%) of these reported zero welfare seizures every year, revealing that only 36 (17.2%) LAs in all countries seized dogs for this reason (255 dogs taken in during 2021; 227 in 2022; and 183 in 2023). The mean number of dogs taken in for welfare reasons per year, per LA (n = 209) was, therefore, low (1.2, 1.1, and 0.9, respectively), but ranged from 0–49 (2021), 0–57 (2022), and 0–25 (2023). Whilst welfare seizures decreased between 2021 and 2023 overall, there were varying patterns between countries (see Table 1).

Data about the seizure of dogs for human safety were provided by 211 (83.1%) of the 254 LAs for all three years, but again, almost all of these respondents reported zero seizures in consecutive years (n = 201, 95.3%). Overall, the number of dogs seized due to danger to humans was low, totalling 67 over the three years, from just 10 LAs. The ROI made the most seizures for safety with 12 in 2021, 14 in 2022, and 20 in 2023.

### 3.3. Breed Types Entering Shelter Accommodation

Of the 254 LAs who provided data for the survey, 156 (61.4%) gave information about the top three breeds entering their kennels between 2021 and 2023. Breed data were categorised as described in the Methods and are summarised in Table 2 alongside the proportion of times each breed category appeared in the top three entering LA kennels. Where a breed type occurred a high number of times, it was given its own category, e.g., Staffordshire Bull Terrier (SBT) and Jack Russell Terrier (JRT), with separate categories created for remaining bull and terrier breeds. Breeds of similar types with small numbers of some breeds within their category were combined into a single category, e.g., ‘Toy’, ‘Sighthound’, ‘Retriever’, and ‘Bulldog’. Breeds that did not appear in the top three more than ten times over the years 2021 to 2023 but did not fit into another similar breed category were combined into ‘Other’ (this included Cane Corso, Mastiff, Rottweiler, Dachshund, Lhasa Apso, Akita, and a ‘restricted breed’). Furthermore, dogs that were described as single crosses were combined with the main breed type category (e.g., SBT and SBT-cross, JRT and JRT-cross, and Labrador and Labrador-cross in the Retriever group). Sometimes LAs reported equal numbers of more than one breed type in first, second, or third place, so these instances were defined as ‘no predominant breed’.

**Figure 1:**
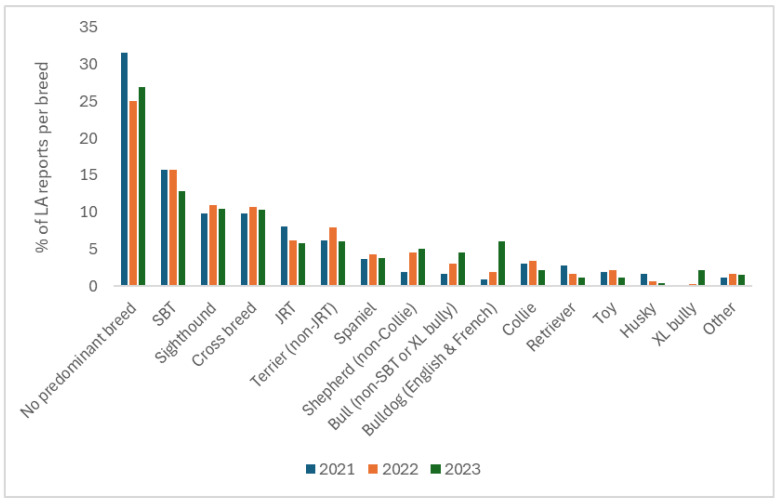
The percentage of times that each breed type was reported by Local Authorities (LAs) (n = 156) as being in the top three breeds to enter their kennels per year (2021–2023) across all countries combined (England, Scotland, Wales, NI, and ROI).

Almost a third of LA responses (27.8%) were classed as ‘no predominant breed’. Over the three years, Staffordshire Bull Terriers (SBT) were most commonly reported as being in the top three breeds entering LA kennels, accounting for 14.8% of the overall responses. This was closely followed by sighthounds (10.4%) and cross breeds (10.3%), and then Jack Russell Terriers (JRT) (6.7%) and non-JRT Terriers (6.7%). In addition to the number of times that each breed type appeared in the top three overall, SBTs were reported more than any other breed as the first (28.4%) and second (12.2%) most common to enter kennels over the three-year period, whilst the Jack Russell Terrier (9.4%) was most frequently the third most common. Figure 1 shows the proportion of times that each dog breed category was reported in the top three per year across all countries and reveals an increase in XL Bull breeds (0–2.1%), Bull breeds (1.7–4.5%), Bulldog (0.9–6.0%), and Shepherd breeds (1.9–5.1%) from 2021 to 2023, whilst other breed types remained relatively static or decreased in being the most common to be brought into LA shelters.

**Figure 2:**
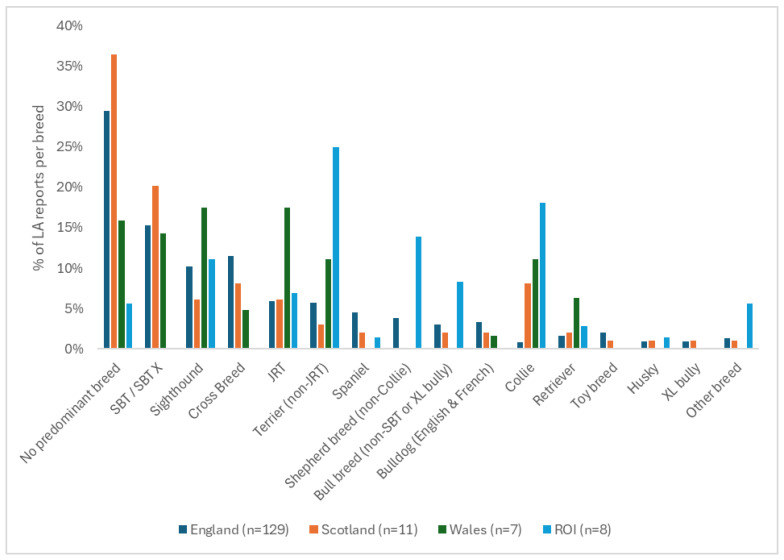
The percentage of times that each breed type was reported by Local Authorities (LAs) (n = 156) as being in the top three breeds to enter their kennels between 2021 and 2023 in England, Scotland, Wales, and ROI. NI not included because n = 1 LA.

Considering the breed data provided per country, response rates from English LAs were highest (67.5%, n = 129), followed by Scotland (58.0%, n = 11), Wales (53.8%, n = 7), and the ROI (36.4%, n = 8), and were lowest from NI (11.1%, n = 1). Figure 2 shows the proportion of times each breed category was reported in the top three per country (except NI, n = 1) for 2021 to 2023 combined. There was variation between countries over which breeds appeared in their top three. In England, the first, second, and third most common breed types were SBT, cross-breed, and sighthound; in Scotland the top three were SBT and equally in second place, cross-breeds and Collies; in Wales, sighthounds and JRTs were jointly most common, followed by SBTs; whilst in ROI, non-JRT Terriers were followed by Collie and other Shepherd breeds.

### 3.4. Length of Stay

A total of 152 (59.8%) LAs provided a response for dogs’ average length of stay in their shelter, with the remaining 102 LAs leaving this question blank or stating data not held. Over a third of these respondents (56, 36.8%) stated a seven-day stay, which corresponds to the UK statutory period that dogs are held by LA kennels before relocating them, e.g., to a rehoming centre. The mean length of stay, including those with a seven-day stay, was 10.8 days (range 1–75 days). Despite having only a five-day statutory period in the Republic of Ireland, their LAs housed dogs for the longest average number of days (23.5 days, range 7–67 days), followed by England (10.4 days, range 1–75 days), Wales (10.1 days, range 6–30 days), Scotland (7.8 days, range 1–23 days), and Northern Ireland (3.7 days, range 3–5 days).

### 3.5. Rehoming

One hundred and ninety-four (76.4%) LAs provided data for all three years on the number of dogs rehomed directly from their shelter, and the same number provided figures on the number of dogs moved elsewhere (defined as moving the dog to another kennels or rehoming NGO where it is no longer the responsibility of the LA), although not all of these provided data for both. The remaining 60 LAs left these questions blank or stated that the data were not held, and the data from one LA was removed as they reported around 300% more dogs were rehomed annually than were taken in, so their response was deemed erroneous.

Of the responding LAs (Table 3), 84 (43.3%) reported no direct rehomings and 24 (12.4%) reported that no dogs were rehomed elsewhere over the 3 years. Across all three years, more than twice as many dogs were moved elsewhere (18,498), compared to dogs rehomed directly by the LA (8906). Overall, the number of dogs who left LA kennels annually via both channels increased year on year, for all countries. The number of dogs moved elsewhere by LAs in England increased by particularly high numbers year on year, from 2191 (2021) to 3442 (2022) to 4370 (2023).

**Figure 3:**
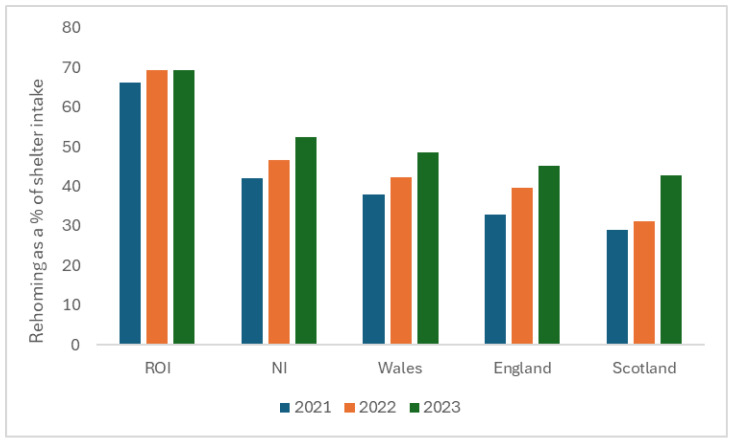
Dogs rehomed expressed as a percentage of shelter intake in LAs (n = 155) across ROI, NI, Wales, England, and Scotland from 2021 to 2023.

The number of dogs that were rehomed in total each year via both channels as a proportion of the shelter intake (n = 155 LAs) also rose from 41% in 2021 to 49% in 2022 to 53% in 2023. This year on year increase was true for all counties (Figure 3), with the ROI (n = 22) rehoming the most dogs in proportion to intake each year with an overall mean across the three years of 68.3%, followed by NI (n = 9) with 47.0%, Wales (n = 8) with 43.0%, England (n = 104) with 39.2%, and Scotland (n = 12) rehoming 34.4%.

### 3.6. Euthanasia Frequency

Local Authorities were asked to provide the number of dogs euthanased over the three years by breed. Of the 254 LAs who responded to the FOI request, 218 (85.8%) provided numerical euthanasia data for each year. Of these, 187 (85.8%) reported euthanasia and 31 (14.2%) stated no occurrences of euthanasia across all three years. The breakdown of euthanasia numbers per year, per country is summarised in Table 4.

**Figure 4:**
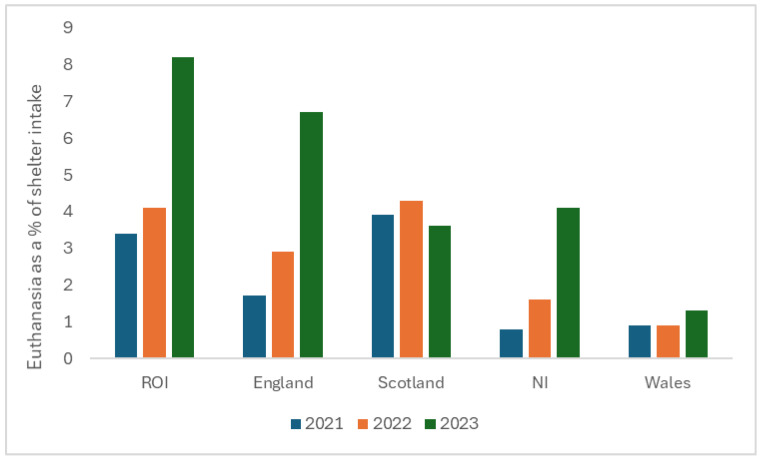
Dogs euthanased, expressed as a percentage of shelter intake in LAs (n = 198) across ROI (n = 22), England (n = 142), Scotland (n = 16), NI (n = 9), and Wales (n = 9) from 2021 to 2023.

The total number of dogs reportedly euthanased across all LAs vastly increased year on year, with numbers more than doubling from 2021 (308) to 2022 (665) and then again in 2023 (1564). The rise in euthanasia rates appears especially notable in England, Northern Ireland, and the Republic of Ireland, with numbers in Scotland and Wales increasing less dramatically. Since the intake of dogs also increased year on year (Table 1), euthanasia figures are reported as a proportion of the intake for those LAs who provided data for both in Figure 4 (n = 198). In all countries combined, 1.9% of LA kennelled stray dogs were euthanased in 2021, compared with 3.0% in 2022 and 6.3% in 2023. The greatest proportion of dogs to be euthanased over all three years occurred in the ROI (5.6%, n = 22), followed by England (4.1%, n = 142), Scotland (3.9%, n = 16), NI (2.4%, n = 9), and Wales (1.1%, n = 9). When considering the proportion of kennelled dogs euthanased per country by year, it varied only marginally over time for Wales and Scotland, whereas in England, NI, and the ROI, the increase in reported euthanasia was approximately five times greater between 2021 and 2023 in NI, four times in England, and twice as great in the ROI. Of particular concern, 8.2% of dogs taken into shelter were euthanased in ROI in 2023, 6.7% were euthanased in England, 4.1% in NI, 3.5% in Scotland, and 1.3% in Wales in the same time period.

### 3.7. Euthanasia by Breed

Of the 187 LAs who reported that euthanasia had been conducted between 2021 and 2023, 134 (71.7%) included information about breeds each year. Breed types were categorised for reporting, as shown in Table 5, with some differences in categorisation compared with the top three breeds to enter kennels (Table 2) because of the higher variation in the breed types euthanased and higher numbers of some breeds for euthanasia compared with shelter entry. For example, Mastiff breeds were given their own category for euthanasia, whereas they appeared in ‘Other’ in the top three breeds to enter kennels. Where named breeds were reportedly euthanased less than 10 times on all three years, they were combined within a broader breed type category where possible, e.g., the ‘Spaniel’ category consisted of English Springer, Cocker, and King Charles Spaniels, and any dogs described simply as ‘Spaniel’ by the LA, or placed as ‘Other’ if there was no clear breed-type category for them.

**Figure 5:**
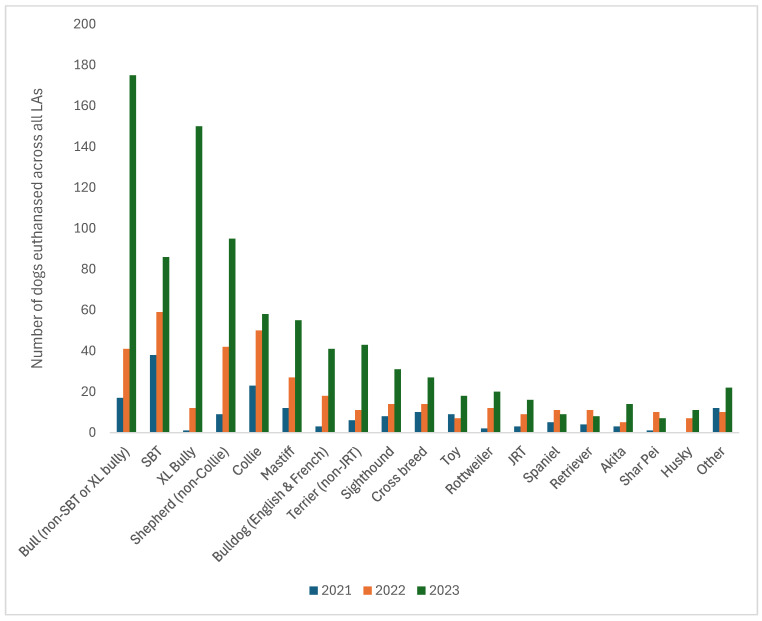
Numbers of individual dogs euthanased from all LAs (England, Scotland, Wales, NI, and ROI) (n = 134) by breed category from 2021 to 2023.

Combined data from the 3 years across all countries revealed that bull-type breeds were euthanased more than any other breed, with Bull breeds, Staffordshire Bull Terriers, and XL Bullies put to sleep first, second, and third most commonly overall (Figure 5). There were increases in the euthanasia of all breeds between 2021 and 2023, but this was especially the case for all the bull breed categories. In 2021 and 2022, Staffordshire Bull Terriers (SBT) were euthanased more than any other breed. However, in 2023, they were fourth most-commonly euthanased, with the bull breed category being most commonly euthanased, followed by XL Bullies and non-Collie Shepherd breeds.

### 3.8. Euthanasia and Welfare Policies

Of all 254 LAs, only 59 (23.2%) reported that they had a written policy for euthanasia and 68 (26.8%) stated they had one for welfare, with just 45 (17.7%) holding a policy for both. High numbers stated they did not have a written euthanasia policy (136, 53.5%) or welfare policy (129, 50.8%). The remainder either did not provide an answer or said that they did not hold any information about welfare or euthanasia policies. LAs who outsource their dog shelter services (e.g., via rehoming organisations) may not have described policies adhered to by the establishment. Of the 187 (85.8%) LAs reporting euthanasia between 2021 and 2023, only 48 (25.7%) stated that they had a written euthanasia policy. A mean number of 17.5 dogs were euthanased over the three-year period per LA who had a euthanasia policy (n = 48), whilst 12.1 dogs were euthansed per LA by those who either stated they did not have a euthanasia policy, left the question blank, or stated data not held (n = 139). It was not possible to conduct statistical analysis to compare the mean number euthanased with/without policies per country, given the low number of LAs who had a euthanasia policy and conducted euthanasia.

### 3.9. Staff Numbers and Training

Sixty-two (24.4%) respondents provided numerical data about the number of Full-Time-Equivalent (FTE) staff working at their kennel service provision. However, one response from a Scottish LA was considered erroneous (58 FTE staff for an average of 20 dogs/year) and, therefore, removed, leaving 61 (24.0%) responses. Not all LAs explicitly stated full- or part-time staff members, so they were assumed to be full-time unless stated part-time (recorded as 0.5 FTE per person). The number of FTE staff across all LA kennels (n = 61) ranged from 0.3 to 13.5, with an average of 4.3 (Wales, 8.3; England, 4.8; ROI, 2.9; NI, 2; and Scotland, 3.2). Notably, 76 (30%) LAs reported zero members of staff, some of which stated that they used animal welfare charity kennels (e.g., Battersea, Dogs Trust, the RSPCA, or smaller local providers). The majority of the remaining 108 LAs advised that they did not hold data on staff numbers, while others advised their service was contracted to a 3rd party or left the question blank.

Of the 61 LAs who provided FTE staff numbers, 46 also gave an answer about staff training. However, only 18 LAs said that their staff had undergone formal further (Level 1–3; equivalent to school-level qualification) or higher education (Level 4–8; post-school college/university) in an animal-related discipline: 11 LAs had staff who were educated up to or equal to Level 3; five LAs had staff with Level 4, 5, or 6 qualifications; and two LAs had staff with an MSc or PhD (Level 7 or 8). Of the remainder, six LAs said that their staff had no formal qualifications and six said that their staff received training, but this was unspecified. Staff at four LA kennels received ‘on the job’ training in animal-related topics (e.g., dog handling, first aid, canine health, and animal welfare), but the level was unspecified. Seven LAs stated that their staff had a qualification, but did not provide the subject or level. Three LAs said they had an attending vet or vet nurse, but their kennel staff training was unstated. Finally, two LAs simply stated that their staff training or education was unknown.

## 4. Discussion

This FOI request to Local Authorities in the UK and ROI revealed that the number of dogs entering shelter accommodation increased in all countries year on year from 2021 to 2023, alongside a disproportionate increase in the number of dogs being euthanased in all countries except Scotland. Over the three-year period, euthanasia doubled as a proportion of the intake in the ROI (to a total of 8.2% of the intake), quadrupled in England (to 6.7% of the intake), and increased fivefold in NI (to 4.1% of the intake).

The reported rise in homeless dogs in the UK and ROI during the three years mirrors the sudden increase in estimated stray dogs in the UK Dogs Trust Stray Dog Surveys over the same time period [6] and reported for the ROI [7]. The Dogs Trust suggested that the rise may be related to the Cost of Living Crisis in the UK, in which dog owners may be disproportionately affected by ‘Dogflation’ [6]. Dog owners are increasingly considering cheaper options for food and other essentials as well as skipping non-emergency veterinary care and record numbers of owners are making enquiries about giving up their dog [6]. Furthermore, between 2021 and 2022, the RSPCA reported a 6% increase in dogs taken into their care alongside decreasing adoption rates and have raised their concerns of a potential rehoming crisis [13]. An increase in shelter-living dogs presents concerns, since it is widely reported that the kennel environment can compromise emotional health [14,15,16,17,18], especially over increasing periods of time [19,20,21]. Certain characteristics of kennel conditions can make it difficult for dogs to cope, such as a loss of social attachments and limited social contact with people and dogs, the kennel size, and the restriction of movement and exercise and disruptive sounds [22,23]. Furthermore, dogs living for prolonged periods in the shelter environment may be more likely to develop behaviour problems [4,5], which not only impact welfare, but may affect their length of stay and adoption success [24], and increase the risk of euthanasia [24,25,26,27,28], where 50–70% of euthanasia in animal shelters is linked with behaviour [26,29]. Kennelling stray dogs also presents significant financial costs to LAs and private organisations for the maintenance and upkeep of dogs [30], impacting directly and indirectly on surrounding communities.

Relatedly, recent interviews with a selection of dog owners who were accessing support from the welfare and rehoming charity Blue Cross, highlighted that economic vulnerability can lead to difficult situations with people’s pets and increasing owner stress and anxiety, with all participants alluding to the expense of having a pet and rising costs in all aspects of their care [31]. The authors also found that members of the public can be reluctant to seek support for their pets, through fear of being judged, disclosure, and stigma, with the welfare implications of this including no access to veterinary, nutritional, or behavioural services and increased cases of abandonment [32]. There is currently an increased prevalence of behavioural issues in the UK pet dog population [33], which is likely to have been exacerbated by the COVID-19 pandemic [33,34] due to social isolation for more mature dogs [34] and the rise in ‘Pandemic Puppies’ [35,36]. Indeed, puppy acquisitions motivated by the pandemic have been associated with an increase in ‘Perceived Costs’ to owners, predominantly due to behavioural risk factors such as aggression and separation-related problems [37]. The COVID-19 pandemic may have had an impact on the number of dogs entering LA shelters in the UK and ROI during our study period, with behavioural problems being a leading cause for relinquishment [38] and, potentially, abandonment.

Our FOI request revealed that there were some clear differences in reported breeds entering LA shelters between countries, with Staffordshire Bull Terriers most commonly in the top three in England, Wales, and Scotland, but not in the ROI, where other Terrier and Collie breeds were most likely to be in the top three. Whether the prevalence of breeds in each country, or cultural differences around breed use, could impact their likelihood of entering a shelter are hypotheses that may be worthy of further investigation. It is also important to acknowledge that the accuracy of the LA reporting on breeds or cross-breeds is likely to be flawed. As is the case for the majority who work with dogs of an unknown heritage (e.g., vets, behaviourists, rehoming organisations, and trainers), shelter staff must rely on physical appearance to identify dog breeds. Visual identification is known to be challenging and unreliable even for experienced stakeholders, particularly for cross-breeds [39,40]. For example, only around a quarter of canine experts were able to correctly identify the prominent breed in mixed breeds based on physical characteristics, measured against genetic breed profiles [41].

Unreliable breed identification can have irreversible consequences for welfare outcomes, particularly where breed restrictions and decisions about euthanasia are concerned. In the current study, the reported proportion of dogs euthanased year on year increased across all countries, except Scotland. The three most common breed types reportedly euthanased over the three years were different bull breed types, with the most startling increase in euthanasia for dogs reported as XL bullies, from 1 case in 2021 to 150 dogs in 2023. With the 2023 change in legislation to the Dangerous Dogs Act 1991 [42], restricting XL bull-type dogs in England and Wales, and with similar changes following in Scotland [43,44] and the ROI in 2024 [45], it is plausible to consider that the rising euthanasia of bull breeds in LAs may be related. The Dogs Trust recently reported that the proportion of dogs euthanased by LAs in the UK due to the Dangerous Dogs Act rose from 7% in 2023 to 24% in 2024, concluding that this was likely to be at least in part related to the ban on XL bully-type dogs [46]. Our data also reflected previous work that has shown that larger dogs, bull breeds, and brachycephalic dogs are harder to rehome and are often associated with longer stays in shelters and higher euthanasia rates [47,48,49]. Staffordshire Bull Terriers—the most common breed type reported to enter LA kennels between 2021 and 2023—are commonly misclassified as dangerous dogs [39] and inappropriately destroyed, or otherwise remain kennelled long-term due to dog breed stereotypes impacting their adoptability [50]. As aforementioned, dogs with behavioural issues are also at higher risk of relinquishment into shelters and may be more likely to be euthanased [24].

The proportion of dogs taken into shelter by the UK and ROI LAs that were then rehomed directly or via a rehoming organisation increased during the study period (2021 to 2023) in all countries. Whilst our study did not capture the number of dogs reunited with their owners after having been handled by LA kennels, it can be assumed that the majority who were not euthanased or rehomed were reunited, and in the ROI, this was reported as 19% in 2023 [2]. Interestingly, in their most recent estimates for 2024, the Dogs Trust reported that more dogs were passed to welfare organisations by LAs than were reunited with their owners [46]. In the same year they also reported another upsurge in the number of dogs being handled by LAs and the proportion that were rehomed and euthanased.

Given the recent rise in the number of dogs who enter shelter kennels and the increase in euthanasia rates, it is also concerning that we found an apparent lack of welfare and euthanasia policies and low numbers of staff with formal qualifications relating to animal welfare. The Association of Dog and Cat Homes Minimum Welfare and Operational Standards requires competency of staff through training, stating “People should hold a relevant qualification… and/or be able to clearly evidence knowledge through experience” [51]. Whilst this provides for experiential learning, for example, shadowing senior staff, there is a recognition of the usefulness of formal training and qualifications. These results highlight areas for improvement in welfare and education in this sector.

This FOI request to LAs resulted in a response rate of 63%, who provided a full or partial dataset for all three years. Whilst FOI legislation enforces transparency by binding public authorities to respond to information requests [52], a lack of compliance with FOI laws has been highlighted as a concern [53]. Public authorities can refuse to provide data under 23 circumstances of exemption (for example, if it is deemed too great a financial cost, for the protection of law enforcement interests, or if it would go against public interest). However, in this study, 20.8% of LAs failed to either acknowledge or respond to the FOI request. Therefore, we acknowledge the issue of non-responders and a lack of information about whether LAs with poorer welfare outcomes, higher euthanasia rates, or weaker record-keeping systems are under-represented within the dataset. Whilst some LAs gave statements of exemption or calculated a prohibitive cost to providing information, measuring the influence of a non- or partial response upon the reported results, or any systematic difference between responding and non-responding Las, is outside the reach of this study. This highlights a limitation of information gathered by FOI requests, which have the potential to lead to a biased study population. A recent study exploring local government responses to FOI requests in Slovakia gained an overall response of 47.5% and also found that the probability of gaining a response increased by 23% with less demanding queries [54]. FOI requests can also elicit excellent response rates, where two studies of medical education in universities both gained 100% FOI request responses [55,56].

A lack of consistency was sometimes seen in the current study about what information responding LAs were able or willing to access and share. For example, some provided information despite outsourced kennel provisions, whereas others claimed data were not held or could not be provided due to outsourcing. Some responses were unclear, for example, some LAs gave annual intake figures that exceeded the number of dog spaces that they stated were available at any one time, and others provided numbers of staff that exceeded the number of dogs. Clearly erroneous responses were removed (e.g., an instance where the number of staff was 300% higher than the number of dogs), yet fewer outlying answers were retained, as we could not speculate their accuracy. Whilst ambiguous data may be due to errors in the LA data storage, the response, or question interpretation, in these given examples, it could be that the LA shelter kennels were embedded within a larger rehoming organisation and their data were not separated, leading to less reliable responses. The lack of consistency and the ambiguity in some responses highlight some wider constraints of FOI research. The FOI Act does not give a right to high-quality information, rather, researchers rely upon existing information held by public bodies and are limited by the data collection, storage, and extraction processes of both the organisation and individual responding to the request, limiting the possibility of ascertaining the comprehensiveness or accuracy of data provision [57].

A low number of LAs reported that dogs entered their shelters after being seized for human safety (10 LAs) or animal welfare (36 LAs), with many others advising that the responsibility for these dogs falls largely to welfare charities or the police. Another area that we asked LAs about was relinquishment, and some explained that they did not provide a service for unwanted dogs (with this being the responsibility of rehoming charities). However, some LAs who provided data for the relinquishment question described it as the number of dogs reclaimed by their owners. These answers were deemed erroneous and excluded from the dataset, but it is possible that some reclaim data were given instead of relinquishment figures without being explicitly described, which may have, therefore, been inadvertently included in our relinquishment data. Such issues suggest that FOI requests would benefit from more explicit requests, with a rigid answer template to help reduce or limit erroneous or ambiguous responses. The less clear outcomes highlighted in our current study warrant further investigation.

The dog populations of the UK and ROI are closely linked, with the ROI having a strong sector breeding puppies for export, predominantly to the UK. For example, in 2016 the Dublin SPCA estimated 70–80,000 puppies moving from the ROI to the UK, both legally and illegally, accounting for around 10% of all puppies sold in the UK [58]. In addition, the cultural similarities between Ireland and the UK suggest that changes in one country are often mirrored in the other. Some LAs provide accessible summary statistics (NI) or report (ROI) on a wide range of topics relating to dog control and its impact on the community (e.g., dog licences, complaints about stray dogs, dog attacks, dogs exiting shelters, and dog warden enforcement actions). However, given the large variation in LA responses to this FOI request and in how their data were held (often inaccessibly), we recommend a nationally available resource of harmonised stray dog statistics that would enable monitoring over time and between LAs, regions, and countries and provide opportunities to identify ways of reducing the number of dogs entering shelter kennels to improve welfare and reduce public spending. This should include data on the intake and outcomes, including granularity by breed. In addition, we recommend that all LA shelters have a formal animal welfare policy that includes euthanasia decisions and that, for societal transparency, these should be publicly available.

## 5. Conclusions

This study provides an overview of dogs entering Local Authority kennel facilities across the United Kingdom and Republic of Ireland between 2021 and 2023, collated from FOI request data. Most notably, we have reported the sustained rise in the number of dogs entering LA shelters and a concurrent yet disproportionate increase in euthanasia rates, with variation between countries, alongside a lack of formal written euthanasia or welfare policies. Our study revealed several potential negative consequences of recent legislative changes and breed restrictions. Directing resources to effectively promote and support responsible dog ownership may be helpful to ensure compliance with legislation and prevent further escalation of euthanasia numbers and dog abandonment. Considerable inconsistencies between LA responses to the FOI request and in, e.g., data recording, reporting practices, and the presence of welfare and euthanasia policies highlight systemic challenges faced by LAs and for those wishing to access data that should be publicly available. Our recommendation of a nationally available resource of harmonised summary data for stray dog statistics across the UK and ROI, would be a step towards an improved, standardised understanding and more cohesive and transparent management of the stray dog population, which should have positive outcomes for animal welfare and financial costs.

## Figures and Tables

**Table 1:** The number of dogs reported by Local Authorities (LAs) as entering their shelter kennels in total per country (England, Wales, Scotland, Northern Ireland [NI], and Republic of Ireland [ROI]) per year (2021, 2022, and 2023) and for each reason for intake (stray; relinquishment; welfare seizure; and human safety seizure) per country, per year. Total mean and range data per LA by country and year is also provided.

	Year	England (n = 169)	Wales (n = 11)	Scotland (n = 17)	NI (n = 9)	ROI (n = 22)	Total (Mean, Range per LA) (n = 228)
Total number of dogs entering all LA kennels per year, per country	2021	10,408	1115	363	1685	2739	16,310 (71.5, 0–608)
	2022	14,021	1489	541	2252	4648	22,580 (99.0, 1–638)
	2023	15,532	1707	590	2443	4871	24,781 (108.7, 0–742)
Total number of dogs per country entering LA kennels between 2021 and 2023 (mean and range)		40,368 (78.7, 0–742)	4363 (132.2, 4–638)	1494 (29.3, 0–190)	6380 (236.3, 70–569)	12,258 (185.7, 19–523)	63,671 (93, 0–742)
		England (n = 190)	Wales (n = 13)	Scotland (n = 19)	NI (n = 9)	ROI (n = 22)	Total (n = 253)
Total number of stray dogs per year, per country	2021	11,961	883	404	1662	1612	15,907 (65.3, 0–608)
	2022	15,870	1190	517	2178	2980	22,735 (89.9, 1–535)
	2023	17,785	1461	575	2367	3473	25,661 (101.4, 0–742)
Total number of dogs per country entering LA kennels as strays between 2021 and 2023 (mean and range)		45,616 (80.0, 0–742)	3534 (90.6, 4–372)	1496 (26.2, 0–123)	6207 (229.9, 70–569)	8065 (122.2, 10–453)	64,918 (85.5, 0–742)
		England (n = 134)	Wales (n = 11)	Scotland (n = 14)	NI (n = 9)	ROI (n = 22)	Total (n = 186)
Total number of dogs relinquished per year, per country	2021	407	119	24	329	874	1753 (9.2, 0–192)
	2022	541	166	56	564	1266	2593 (13.6, 0–304)
	2023	507	148	77	736	1181	2649 (13.9, 0–386)
Total number of dogs per country relinquished to LA kennels between 2021 and 2023 (mean and range)		1455 (3.6, 0–132)	433 (13.1, 0–117)	157 (3.7, 0–47)	1629 (60.3, 0–386)	3321 (50.3, 0–157)	6995 (12.3, 0–386)
		England (n = 157)	Wales (n = 12)	Scotland (n = 16)	NI (n = 2)	ROI (n = 22)	Total (n = 209)
Total number of dogs seized on animal welfare grounds per year, per country	2021	98	15	3	63	76	255 (1.2, 0–49)
	2022	84	12	2	10	119	227 (1.1, 0–57)
	2023	80	13	0	19	71	183 (0.9, 0–25)
Total number of dogs per country entering LA kennels as welfare seizure cases between 2021 and 2023 (mean and range)		262 (0.6, 0–36)	40 (1.1, 0–15)	5 (0.2, 0–3)	92 (15.3, 5–47)	266 (4.0, 0–57)	665 (1.1, 0–57)
		England (n = 158)	Wales (n = 12)	Scotland (n = 18)	NI (n = 9)	ROI (n = 14)	Total (n = 211)
Total number of dogs seized for human safety per year, per country	2021	0	0	0	0	12	12 (0.1, 0–7)
	2022	2	0	1	0	14	17 (0.1, 0–12)
	2023	12	2	0	4	20	38 (0.2, 0–9)
Total number of dogs per country seized as human safety cases between 2021 and 2023 (mean and range)		14 (0.03, 0–7)	2 (0.1, 0–2)	1 (0.01, 0–1)	4 (0.1, 0–4)	46 (1.1, 0–12)	67 (0.1, 0–12)

Note: Each category of data was provided separately by Local Authorities and the total number of dogs entering the kennels did not always equal the sum of the four categories of entries.

**Table 2:** Breed categories and descriptions used to summarise Local Authority (n = 156) reports of the top three breeds entering their kennels between 2021 and 2023, along with the proportion of instances that each breed category was reported in the top three breeds.

Breed Category	Description	Proportion of Instances in Top 3 Between 2021 and 2023
No predominant breed	Breed info provided, but in equal numbers	27.8%
Staffordshire Bull Terrier (SBT)	SBT and SBT X	14.8%
Lurcher and sighthounds	Lurcher, Greyhound, Whippet, and Saluki	10.4%
Cross-breed	All non-single breed crosses or those described as cross-breeds by LA	10.3%
Jack Russel Terriers (JRT)	All JRTs and JRT-cross	6.7%
Terrier	Other Terrier breeds (except Yorkshire—see Toy breed)	6.7%
Spaniel	English Springer, Cocker, and King Charles Spaniel, any described as ‘spaniel’ by LA	3.9%
Shepherd breed	German Shepherd Dog and other non-Collie shepherd breeds	3.8%
Bull breed	American Bull Terrier, English Bull Terrier, Boxer, Pit Bull Terrier, and any described as ‘bull breed’ by LA	3.1%
Bulldog	French Bulldog, English Bulldog, and any described as ‘bulldog’ by LA	2.9%
Collie	All Collie breeds	2.8%
Retriever	Labrador Retriever and other Retrievers	1.9%
Toy	Yorkshire Terrier, Bichon Frise, Chihuahua, Shih Tsu, Pomeranian, and Pug	1.7%
Husky	Husky breeds	0.9%
XLBully	XL Bull breeds	0.8%
Other breed	Cane Corso, Mastiff, Rottweiler, Dachshund, Lhasa Apso, Akita, and ‘restricted breed’. Restricted breed refers to breeds with additional legal restrictions (such as being muzzled and on a lead in public) in the ROI, consisting of American Pit Bull Terrier, English Bull Terrier, Staffordshire Bull Terrier, Bull Mastiff, Dobermann Pinscher, Rottweiler, German Shepherd, Rhodesian Ridgeback, Japanese Akita, Japanese Tosa, and ‘Ban Dog’ (or any cross or mixture of the restricted breeds)	1.4%

**Table 3:** The number of dogs reported by Local Authorities (LAs) in England, Scotland, Wales, Northern Ireland (NI), and the Republic of Ireland (RoI) as being rehomed either directly from the LA or moved to another rehoming centre.

		England (n = 139)	Scotland (n = 14)	Wales (n = 10)	NI (n = 9)	ROI (n = 22)	Total (n = 194)
Number of dogs rehomed directly from LA	2021	1235	52	277	56	670	2012
	2022	1681	95	378	106	1225	3485
	2023	1969	152	498	114	1383	4116
Total		4885	299	1153	276	3278	8906
Number of dogs moved to another rehoming centre	2021	2191	35	105	593	1144	4068
	2022	3442	49	182	943	1997	6613
	2023	4370	69	213	1168	1997	7817
Total		10,063	153	500	2704	5138	18,498

**Table 4:** Reported number of dogs euthanased by Local Authorities (n = 218) per country (England, Wales, Scotland, NI and ROI), per year (2021 to 2023).

	England (n = 159)	Wales (n = 11)	Scotland (n = 17)	NI (n = 9)	ROI (n = 22)	Total (n = 218)
2021	177	9	14	15	93	308
2022	404	12	22	37	190	665
2023	1021	18	22	101	402	1564
Total	1602	39	58	153	685	2537

**Table 5:** Breed categories and category descriptions used to summarise Local Authority (n = 134) reports of breeds euthanased between 2021 and 2023.

Breed Category	Description
Bull breed	American Bull Terrier, English Bull Terrier, Pit Bull Terrier, Pocket bulldog, Boxer, any described as ‘bull breed’ by LA
Staffordshire Bull Terrier (SBT)	SBT and SBT X
XL Bully	XL Bull breed
Cross-breed	All non-single breed crosses or those described as cross-breeds by LA
Shepherd breed	German Shepherd Dog and other non-Collie shepherd breeds (Belgian, Kangal, Caucasian, Dutcher Herder)
Collie	Border Collie and other Collie breeds
Mastiff	Mastiff, Bull Mastiff, Presa Canario mastiff, Cane Corso Mastiff, Dogue de Bordeaux, Boerboel, Great Dane
Bulldog	French Bulldog, English Bulldog, and any described as ‘bulldog’ by LA
Terrier	Non-Jack Russell Terrier breeds (except Yorkshire Terrier—see Toy breed)
Sighthound	Lurcher, Whippet, Greyhound, Saluki
Cross-breed	All non-single breed crosses, or those described as cross-breeds by LA
Toy breed	Yorkshire Terrier, Bichon Frise, Chihuahua, Shih Tsu, Pomeranian, Maltese, and Pug
Rottweiler	Rottweiler
JRT	Jack Russell Terrier
Spaniel	English Springer, Cocker, and King Charles Spaniel, any described as ‘spaniel’ by LA
Retriever	Labrador Retriever and other Retrievers
Akita	Akita
Shar Pei	Shar Pei
Husky	Husky
Other	Doberman, Dalmatian, Dachshund, Lhasa Apso, Chow, St Bernard, Weimaraner, Norwegian Ridgeback, Poodle, Alaskan Malamute, Pointer, ‘hound’, Fox Hound, ‘restricted breed’ (11 breeds in ROI with additional legal restrictions)

## Data Availability

Dataset available on request from the authors.
